# A fast stochastic framework for automatic MR brain images segmentation

**DOI:** 10.1371/journal.pone.0187391

**Published:** 2017-11-14

**Authors:** Marwa Ismail, Ahmed Soliman, Mohammed Ghazal, Andrew E. Switala, Georgy Gimel’farb, Gregory N. Barnes, Ashraf Khalil, Ayman El-Baz

**Affiliations:** 1 Bioengineering Department, University of Louisville, Louisville, KY, United States of America; 2 Department of Electrical and Computer Engineering, Abu Dhabi University, Abu Dhabi, United Arab Emirates; 3 Department of Computer Science, University of Auckland, Auckland, New Zealand; 4 Department of Pediatrics, University of Louisville, Louisville, KY, United States of America; Center for Neuroscience and Regenerative Medicine, UNITED STATES

## Abstract

This paper introduces a new framework for the segmentation of different brain structures (white matter, gray matter, and cerebrospinal fluid) from 3D MR brain images at different life stages. The proposed segmentation framework is based on a shape prior built using a subset of co-aligned training images that is adapted during the segmentation process based on first- and second-order visual appearance characteristics of MR images. These characteristics are described using voxel-wise image intensities and their spatial interaction features. To more accurately model the empirical grey level distribution of the brain signals, we use a linear combination of discrete Gaussians (LCDG) model having positive and negative components. To accurately account for the large inhomogeneity in infant MRIs, a higher-order Markov-Gibbs Random Field (MGRF) spatial interaction model that integrates third- and fourth- order families with a traditional second-order model is proposed. The proposed approach was tested and evaluated on 102 3D MR brain scans using three metrics: the Dice coefficient, the 95-percentile modified Hausdorff distance, and the absolute brain volume difference. Experimental results show better segmentation of MR brain images compared to current open source segmentation tools.

## Introduction

**Fig 1:**
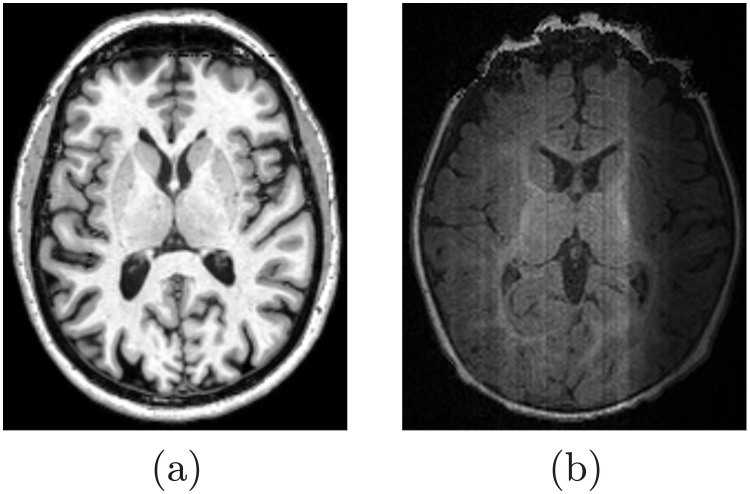
T1-weighted MRI for adult (a) and infant (b) brains.

Accurate delineation of brain tissues from magnetic resonance (MR) images is an essential step in human brain mapping and neuroscience [1–3]. However, brain MRI segmentation faces challenges stemming from image noise, magnetic field inhomogeneities, artifacts such as partial volume effects, and discontinuities of boundaries due to similar visual appearance of adjacent brain structures. This paper addresses brain segmentation from MR images at different life stages, having the infancy stage the most complicated one due to reduced contrast, higher noise [4], and inverse contrast between the white matter (WM) and gray matter (GM) [5], Fig 1. Segmentation of the brain at later stages might be relatively easier, as the contrast between different types of tissue is much better, and the signal-to-noise ratio (SNR) are improved, Fig 1.

Lower contrast between infant brain tissue classes stems from the fact that most of the long axons in the WM are not yet myelinated, and WM water content is close to that of GM. Both the WM and GM have the same average T1-weighted intensity at about 9 months of age [6]; hence, it is difficult to classify the infant brain tissues using only the intensity. Furthermore, the unique challenge of imaging the infant patient precludes techniques that might improve contrast or signal-to-noise ratio (SNR) but would also lengthen the time of acquisition. [2]. Many segmentation techniques have been developed for the last two decades in order to address the brain MRI segmentation challenges. These techniques can be roughly classified into the following categories: (*i*) probabilistic, or statistical methods, (*ii*) atlas-based methods, (*iii*) hybrid methods that include (i) and (ii), (*iv*) deformable-model based methods, and (*v*) deep-learning based methods.

### Probabilistic segmentation

These algorithms involve prior models that describe the signal distributions of each brain structure. Ng et al. [7] segmented MR brain images using the unsupervised K-means clustering of signals and an improved watershed algorithm. A similar approach by Xue et al. [5] employed a parametric Gaussian density estimation with an expectation-maximization (EM) algorithm and constrained spatial homogeneity of the MR images with a Markov random field (MRF) prior. Partial volume averaging effects have been eliminated by predicting the misclassification (e.g., of an “averaged” CSF and GM into an intensity similar to WM). An automated MRI brain segmentation method by Mayer et al. [8] combined spatial and intensity features into a high-dimensional feature space. An adaptive mean-shift classifier extracted a set of convergence modes, i.e. high-density points of a feature space, being good candidates for intensity-based classification. Brain tissues were classified by an intensity-based mode clustering. This approach was very effective with non-convex clustering. Fang et al. [9] developed a tree metrics (TM)-based graph cut algorithm to segment the MRI brain tissues. After a brain MR image is classified using the TM, the goal labeling is inferred by “tree-cutting”. In contrast to most of conventional iterative methods like the EM-based ones, which produce only locally optimal labeling, this algorithm needs no more than one sweep to generate the globally optimal labeling with respect to the TM. An automated segmentation approach by Ortiz et al. [10] classified the brain tissue with no prior information. The segmentation consisted of feature extraction and classification. Extracted first order (pixel/voxel-wise), second order (pair-wise), moment, and scale-invariant features were classified by growing hierarchical self-organizing maps (GHSOM). Li et al. [11] proposed a 3D MGRF model for the segmentation of brain MR images to avoid the shortcomings of the 2D model which is not able to fully capture the spatial information, especially among the slices. An initial segmentation was first obtained by k-means clustering in order to reduce the extensive computations required by the MGRF model. The Iterated Conditional Modes (ICM) algorithm was finally applied to obtain the optimal solution under maximum a posteriori (MAP) criterion. A non-parametric adaptive mean-shift algorithm was proposed by Janney et al. [12] for brain tissue segmentation. The method clustered the joint spatial-intensity feature space, followed by a phase of intensity-based mode clustering into the brain tissue types. Weber et al. [13] segmented the brain tissues using FSL-FAST free software that is based on K-means clustering which provided initial segmentation, followed by the EM algorithm for bias field correction. In order to speed up the process, parallelization to any eligible parts of the software was applied, which needed some adaptation to the algorithms in order to maintain the accuracy obtained by the software package. Mahmood et al. [14] proposed an unsupervised framework for brain tissue segmentation using a combination of Bayesian-based adaptive mean shift that clustered the tissues in the joint spatial-intensity feature space, and fuzzy c-means that is initialized with a priori spatial tissue probability maps to assign the clusters into three tissue types; WM, GM, and CSF.

Infant brain segmentation using statistical-based methods was also addressed in literature. Automated segmentation of brain structures, such as WM, CSF, central GM (CEGM), and cortical GM (COGM) was conducted by Anbeek et al. [15] using T2-weighted and inversion recovery (IR) MRI of the neonatal brains. Probability maps to segment each brain tissue class with a *K*-nearest neighbor (KNN) classifier using voxel intensities and coordinates as features were constructed manually. A multi-label segmentation process combined the obtained classes. Wang et al. [16] employed a random forest technique to integrate features from different modalities for brain tissue segmentation in infants along with probability maps of GM and CWM. Zhang et al. [17] proposed a deep convolutional neural networks (CNNs) approach for segmenting the neonatal brains from multi-modal MR images, generating the segmentation maps as outputs. The multiple intermediate layers included many operations such as convolution, pooling, and normalization in order to capture the highly nonlinear mappings between inputs and outputs. Moeskops et al. [18] segmented neonatal brains into WM, GM, and CSF using supervised voxel classification.

To recapitulate, statistical-based techniques are generally fast to implement compared to other segmentation methods. However, the fact that actual intensity distributions of brain structures are greatly affected by several factors, such as the unique patient and scanner along with scanning parameters, makes the segmentation hard. Also, due to the similar intensities for the different brain tissue structures of the infant MR brain images, segmentation techniques only based on the intensity remain inaccurate.

### Atlas-based segmentation

Atlas-based approaches have emerged as powerful segmentation tools. These approaches are based on a priori knowledge about brain structures, and treat the segmentation problem as a registration one. Ashburner et al. [19] introduced a generative framework that combined image registration, tissue classification, and bias correction. Their framework incorporated a smooth intensity variation and nonlinear registration with tissue probability maps using mixture of Gaussians. Pohl et al. [20] introduced a Bayesian model for simultaneous segmentation and registration. Their framework tried to exploit complementary aspects of registration and segmentation problems. In order to account for different physiological (patient size and weight) and scanning (scanner type and data acquisition protocol) parameters, Han et al. [21] introduced an intensity re-normalization procedure to adjust the prior atlas intensity model to new input data to overcome the problems stemming from using training data acquired from a different scanner that was used for the test data. Artaechevarria et al. [22] proposed a generalized local weighting voting scheme in which the fusion weights were adapted for each voxel based on local estimation of the segmentation performance. The local weighting voting outperformed traditional global strategies that estimate a single value for the segmentation accuracy for the whole image. Sabuncu et al. [23] proposed an automated, label fusion segmentation technique. In order to capture greater inter-subject anatomical variability, each training data set was individually co-registered to the test data set. Then, a nonparametric probabilistic model was employed to fuse the training labels to compute the final segmentation. Morin et al. [24] presented an atlas-based segmentation framework using random walks that combined registration and labeling propagation steps. They used a generative model to provide pixel label probabilities to improve the segmentation for high-confidence labels. To match the target images with atlas images, they used the Affine-Scale Invariant Feature Transform (ASIFT) [25] and Speeded Up Robust Features (SURF) [26] registration techniques. In order to avoid segmentation errors produced by registration imperfection, Lötjönen et al. [27] introduced an optimized pipeline for multi-atlas brain MRI segmentation. They introduced two approaches that combine multi-atlas segmentation and intensity modeling based on using EM and graph cuts for optimization. First, they registered all atlases to the target data and a majority voting was applied to predict the segmentation of the target image. Then, the segmentation was improved using the intensity modeling as a post-processing step. Lijn et al. [28] introduced a segmentation method based on the combination of spatial features and appearance models. They generated a spatial probability map that was obtained from multiple atlas-target image registrations to implement the spatial model. The tissue appearance was modeled by a KNN classifier using Gaussian scale-space features. Then, a Bayesian framework was used to combine both spatial and appearance models and a graph-cut approach [29] was used for optimization. Ledig et al. [30] introduced a framework for labeling whole brain scans by incorporating a global and stationary MRF to ensure consistency of the neighborhood relations between structures with an a priori defined model.

Segmentation of neonatal brains was also conducted in the literature using atlas-based techniques. Segmentation of axial neonatal brain MRI that combined multi-atlas-based segmentation and supervised voxel classification was proposed, [31], in order to segment eight different tissue classes, namely cortical grey matter (CoGM), unmyelinated white matter (UWM), brainstem, cerebellum, ventricles, cerebrospinal fluid in the extra-cerebral space (CSF), basal ganglia, and myelinated white matter (MWM). Some approaches use longitudinal scans at a late-time-point age, where the contrast is much better between different tissue types, from which probabilistic atlases are constructed to guide segmentation of neonatal images [32, 33]. Cherel et al. [34] employed a subject-specific atlas that is based on manually segmented data for brain tissue classes segmentation. The atlases were incorporated with single-atlas expectation maximization (EM) method. Neonatal brains were segmented into 50 regions by [35], where structural hierarchy along with anatomical constraints were employed. Infant brain segmentation using shape priors was also addressed in the literature [36–38].

Atlas-based segmentation techniques show more accuracy compared to statistical-based techniques. Nevertheless, they are still challenged by atlas selection, combination, and the associated heavy computation time. Another major drawback of atlas-based segmentation algorithms is their dependency on the selected features that will be used to link between the test subject and the prior (training) data used in the construction of the atlas. This may lead to inaccurate segmentation, as signals vary due to many factors such as the patient’s age, and the scanning protocol.

### Hybrid methods

The literature also shows methods that exploited both probabilistic models along with shape prior (atlas-based) ones. Song et al. [39] proposed a probabilistic neural network (PNN) for segmenting the brain MRI. Probability density functions of the brain tissues were estimated from reference vectors generated by a self-organizing map (SOM). To reduce the partial volume averaging effects, weighting factors were added to the summation layer’s patterns in a weighted probabilistic neural network (WPNN) and soft labeling was performed by a supervised Bayesian classifier. Patenaude et al. [40] proposed a method that used manually labeled image training data, where the principles of both the active shape and appearance models were utilized within a Bayesian framework, allowing probabilistic relationships between shape and intensity to be fully used. Serag et al. [41] employed high-dimensional feature vectors for segmenting brain subjects using a sliding window approach along with a multi-class random forest classifier. Wang et al. [42] segmented T1, T2, and diffusion-weighted brain images using a sparse representation of the complementary tissue distribution. Initially, the brain tissue was segmented into different structures using a patch-based technique with a library of multi-modality images, having been aligned with their ground-truth segmentation maps. Then the segmentation was refined by integrating geometric constraints.

### Deformable-model based segmentation

Deformable models have also been employed for brain segmentation in the literature. Angelini et al. [43] introduced a multi-phase level set framework for automated segmentation of brain MRIs. The segmentation of the brain tissues (WM, GM and CSF) was solely based on homogeneity (average grey level) measures. To avoid the need for any prior information and to speed up numerical calculation, a random seed for initialization of the deformable boundaries was used. Colliot et al. [44] proposed a deformable-model based approach that used spatial constraints, represented as fuzzy subsets of the 3D image space, as an external force to control the boundary evolution. To avoid manual selection of the model parameters, a training step was required to estimate the spatial constraints parameters. Miri et al. [45] introduced a topology-preserving deformable model framework for the segmentation of brain MRIs. They employed photometric constraints to guide the deformable model deformations to iteratively reclassify the points located at the evolving boundaries. A deformable model approach for the segmentation of brain regions from MR images was proposed by Liu et al. [46]. The deformable contour was implicitly represented by a set of Wendland’s radial basis functions (RBFs) and was evolved by iterative updates of the locations of the RBFs. Huang et al. [47] introduced an automated, hybrid deformable model framework that integrated both image edge geometry and voxel statistics features to regularize the convergence of the deformable contour. Del Fresno et al. [48] described a hybrid method that combined region growing and deformable models for segmentation of different structures in head MRI and Computed Tomography CT scans. Their approach used a Region-Growing (RG) algorithm to compute an approximation of the objects. This was followed by generating closed and oriented surface meshes to enclose the region of interest. The deformable model method geometry was constructed using the RG-list of boundary voxels generating a hole-free surface mesh. To better detect the structures of interest, the user could select few seeds for RG initial segmentation. Wang et al. [49] proposed a multi-phase level set framework to segment brain MR images with intensity inhomogeneity. They modeled the local image intensities using Gaussian distributions with different means and variances. Then, a variational approach minimized an energy function to compute the means and variances that would guide the contour evolution towards the target boundaries. Bourouis et al. [50] developed a level set framework for segmenting brain tissues. Their framework employed an image registration step and a classification step for the initialization of the deformable boundary. The boundary evolution was controlled by a speed function that accounted for both boundary- and region-based properties. Ciofolo et al. [51] developed an automated framework based on level sets for simultaneous segmentation of multiple structures from brain MRIs. The evolution of each level set was driven by a fuzzy decision system that combined three factors: intensity distribution of the 3D MR volume, the relative position of the evolving contours, and a priori knowledge provided by an anatomical atlas. Wang et al. [52] proposed a multi-layer background subtraction technique with a seed region growing approach which used local texture features represented by local binary patterns (LBP) and photometric invariant color measurements in RGB color space for brain segmentation. Zhao et al. [53] segmented brain tissues using a method adapted from Chan and Vese model, named automatic threshold level set without edges. Thresholds were obtained by fuzzy c-mean algorithm.

Segmentation using deformable models was also exploited in infant brains, [16, 32, 33, 42].

The main advantage of deformable-model based segmentation techniques is the ability to segment connected (non-scattered) objects more accurately than the other segmentation methods. However, the accuracy of this method is based on the accurate design of the guiding forces (statistical, geometric, etc.) as well as the model initialization. A summary for all cited related work is provided in Table 1.

**Table 1:** Summary of brain segmentation related work.

Group	Category	Methodolody
**Probabilistic**	Ng et al. [7]	Unsupervised K means clustering.
	Xue et al. [5]	Parametric Gaussian Density Estimation with Expectation Maximization.
	Mayer et al. [8]	Adaptive mean shift classifier and intensity-based mode clustering.
	Fang et al. [9]	Graph cuts algorithm.
	Ortiz et al. [10]	Growing hierarchical self-organising map (GHSOM) classifier and probability clustering.
	Li et al [11]	K-means clustering and iterated conditional modes.
	Janney et al [12]	Clustering joint spatial-intensity feature space.
	Weber et al [13]	K-means clustering and Expectation Maximization algorithm.
	Mahmood et al [14]	Bayesian framework and fuzzy c-means.
	Anbeek et al [15]	Probability maps and KNN classifier.
	Wang et al [16]	Probability maps and random forest classifiers.
	Zhang et al [17]	Convolutional neural networks.
	Moeskops et al [18]	Supervised voxel classification.
**Atlas-based**	Ashburner et al [19]	Registration.
	Pohl et al [20]	Simultaneous registration and segmentation.
	Han et al [21]	Intensity re-normalization for prior shape.
	Artaechevarria et al [22]	Local weighting scheme from fusion.
	Sabuncu et al [23]	Label fusion.
	Morin et al [24]	Labeling propagation steps.
	Lotjonen et al [27]	Atlas with majority voting and intensity models.
	Van et al [28]	Spatial and appearance models.
	Ledig et al [30]	Markov Random Field and prior shape.
	Srhoj et al [31]	Multiple atlases with supervised voxel classification.
	Makropoulos et al [35]	Structural hierarchy and anatomical constraints.
	Cherel et al [34]	Subject-specific atlas with single-atlas expectation maximization.
**Hybrid**	Song et al. [39]	Probabilistic neural network.
	Patenaude et al [40]	Active shape model within a Bayesian framework.
	Wang et al [42]	Sparse representation of tissue distribution.
	Serag et al [41]	Sliding window and random forest classifier.
**Deformable**	Angelini et al [43]	Multi-phase level set framework.
	Colliot et al [44]	model with spatial constraints.
	Miri et al [45]	Topology-preserving model with photometric constraints.
	Liu et al [46]	Radial Basis Functions.
	Albert et al [47]	Edge geometry and voxel statistics.
	Del et al [48]	model initialized with region growing.
	Wang et al [49]	Multi-phase level set framework.
	Bourouis et al [50]	Level sets initialized by registration.
	Ciofolo et al [51]	Level sets driven by a fuzzy decision system.
	Wang et al [52]	Region growing with local texture features.
	Zhao et al [53]	Automatic threshold level sets without edges.
**DL**	Brebisson et al [54]	3D and orthogonal 2D intensity patches.
	Zhang et al [17]	Multi-modality information.
	Chen et al [55]	low-level image appearance features, implicit shape information, and high-level context.

### Deep-learning based segmentation

De Brebisson et al. [54] proposed a deep artificial neural network for brain image segmentation from MR scans that assigns each voxel to its corresponding anatomical region. The information to the network is obtained at different scales around the voxel of interest: 3D and orthogonal 2D intensity patches capture a local spatial context. Also, large compressed 2D orthogonal patches and distances to the regional centroids are used so that they would enforce global spatial consistency. Zhang et al. [17] proposed a method that is based on deep convolutional neural networks for multi-modality isointense infant brain image segmentation. Multi-modality information from T1, T2, and fractional anisotropy (FA) images were exploited as inputs to generate the segmentation maps as outputs. The multiple intermediate layers applied convolution, pooling, normalization, and other operations to capture the highly nonlinear mappings between inputs and outputs. Chen et al. [55] proposed a deep voxelwise residual network, referred as VoxResNet, which handles volumetric data. It integrated the low-level image appearance features, implicit shape information, and high-level context together for improving the volumetric segmentation performance.

In summary, brain segmentation work found in the literature suffer from many drawbacks as mentioned above. Moreover, infant brain segmentation techniques depend on either multiple modalities which lengthens the processing time, or on longitudinal studies which are not always available for research purposes. Also the reduction in contrast between CWM and other structures hardens the issue of preserving its edges by most of the available techniques.

To overcome the aforementioned limitations, this paper proposes a novel technique for brain segmentation from MR images. Adaptive probabilistic shape models for the shape and first-order visual appearance of MRI data are employed to initialize the segmentation. This is then combined with a novel higher-order Markov Gibbs random field (MGRF) spatial interaction model (up to fourth order) with analytic estimation of potentials. This joint model guarantees increasing the segmentation accuracy by accounting for the large-scale inhomogeneities and noise in infant brain MRI data. Also, the analytic estimation of potentials generalizes the proposed model to different MRI subjects, unlike using empirical values in most of the work present in literature which would require manual setting for each subject. The strength of the proposed algorithm lies in the fact that it neither depends on multiple modalities for acquiring images nor on longitudinal studies.

## Methods

**Fig 2:**
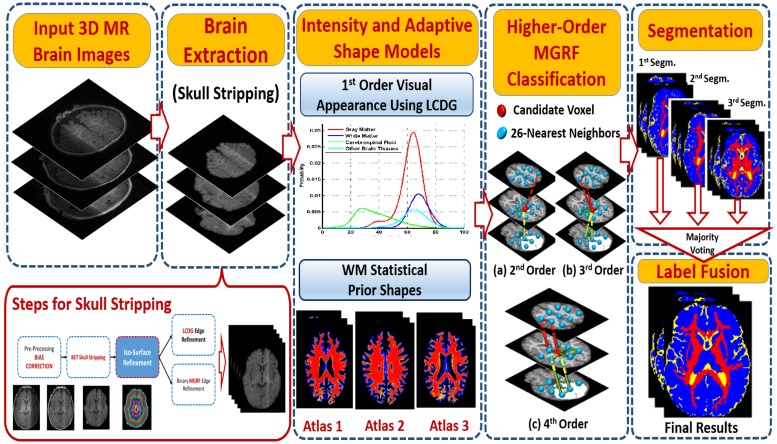
Proposed segmentation framework.

Some brain tissues such as the brain stem and cerebellum are similar to the WM and GM intensities. Therefore, the segmentation technique, Fig 2, makes use of the adaptive shape prior that is co-guided by both a first-order visual appearance descriptor using the estimated LCDG models for each class as well as the 3D spatial relationships between the region labels to segment each label. This forms a 3D joint model that integrates shape, intensity, and spatial information.

### Preprocessing

Before segmentation takes place, bias correction is applied to the brain volumes using the nonparametric approach proposed by Tustison at al. [56] to account for nonuniform intensities. This is followed by applying a 3D generalized Gauss-Markov random field (GGMRF) model [57] that reduces noise effects and removes inconsistencies of the scans.

The skull is then removed before segmentation takes place. This work uses the automated approach proposed by our group in [58], which refines the skull stripping results from the brain extraction tool (BET). This is achieved using an additional processing step that is based on the geometric features of the brain. Since the non-brain tissues are brighter than brain tissue, this step exploits the visual appearance features of the MR brain data. Namely, an evolving iso surface-based approach is proposed to remove the non-brain tissues, which is guided by the MR data visual appearance features. First, a set of nested iso-surfaces are generated by the fast marching level sets (FMLS), using the calculated distance map of the extracted brain from the BET step. Then, classification of voxels as brain or non-brain is conducted. Results on both infants and adults using this approach showed the capability of the proposed approach, outperforming four widely used brain extraction tools: BET, BET2, brain surface extractor (BSE), and infant brain extraction and analysis toolbox (iBEAT).

### Joint MGRF model of MR brain images

Let **R** = {(*x*, *y*, *z*) : 0 ≤ *x* ≤ *X* − 1, 0 ≤ *y* ≤ *Y* − 1, 0 ≤ *z* ≤ *Z* − 1}; **Q** = {0, 1, …, *Q* − 1}; and **L** = {0, …, *L*} denote a finite 3D arithmetic lattice of the size of *XYZ* supporting grayscale images and their region (segmentation) maps, a finite set of *Q* integer gray values, and a set of region labels *L*, respectively. Let **g** = {*g*_*x*,*y*,*z*_ : (*x*, *y*, *z*) ∈ **R**; *g*_*x*,*y*,*z*_ ∈ **Q**} and **m** = {*m*_*x*,*y*,*z*_ : (*x*, *y*, *z*) ∈ **R**; *m*_*x*,*y*,*z*_ ∈ **L**} be a grayscale image taking values from **Q**, i.e., **g** : **R** → **Q**, and a region map taking values from **L**, i.e., **m** : **R** → **L**, respectively. An input brain image, **g**, co-aligned to the training data base, and its map, **m**, are described with a joint probability model: *P*(**g**, **m**) = *P*(**g**|**m**)*P*(**m**), which combines a conditional distribution of the images given the map *P*(**g**|**m**), and an unconditional probability distribution of maps *P*(**m**) = *P*_sp_(**m**)*P*_**V**_(**m**). Here, *P*_sp_(**m**) denotes a weighted shape prior, and *P*_**V**_(**m**) is a Gibbs probability distribution with potentials **V**, which specifies a MGRF model of spatially homogeneous maps **m**. Details of the model’s components are outlined below.

#### Adaptive shape model *P*_sp_(m)

**Fig 3:**
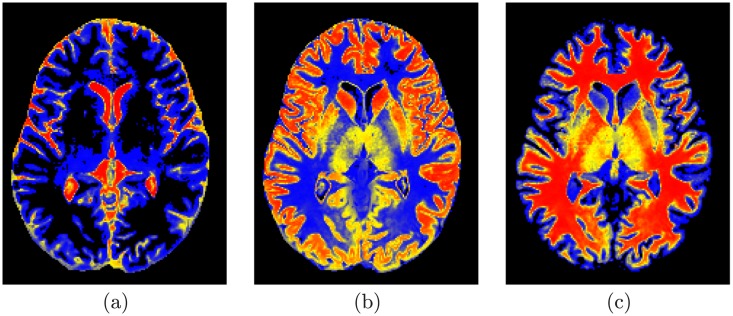
The calculated shape probability for the CSF(a), GM(b), and WM(c).

To start the segmentation process, a database is created, where expected shapes of each brain label are constrained with an adaptive probabilistic shape prior. To create the atlases, a training set of images, collected for different subjects (not included as test subjects), are co-aligned by 3D affine transformations with 12 degrees of freedom (3 for the 3D translation, 3 for the 3D rotation, 3 for the 3D scaling, and 3 for the 3D shearing) in a way that maximizes their Mutual Information (MI) [59]. An atlas of 10 subjects containing the three labels to be segmented (WM, GM, and CSF) was constructed for each age group (infants, children, and adults) as described. For each input MR data to be segmented, the shape prior is constructed by an adaptive process guided by the visual appearance features of the input MRI data [60–62]. The shape prior is a spatially variant independent random field of region labels for the co-aligned data:

$$\begin{array}{c} P_{\text{sp}}\left(m\right)=\prod_{(x,y,z)\in R}p_{\text{sp}:x,y,z}\left(m_{x,y,z}\right) \end{array}$$ (1)

where *p*_sp:*x*,*y*,*z*_(*l*) is the voxel-wise empirical probabilities for each brain label *l* ∈ **L**. First, the normalized cross correlation similarity coefficient is used to select the subject from the shape database that has the best match with the input subject (i.e., highest similarity). The selected subject is then used as a reference prototype to co-align the input subject using the 3D affine transformation described above. In order to estimate the shape prior probabilities for each voxel in the test subject, the steps summarized in Algorithm 1 are followed. Fig 3 show the calculated probabilistic maps for each structure using the proposed adaptive shape prior.

**Algorithm 1** Steps of the shape prior segmentation.

1. Align the test subject with the shape database to get the 3D affine transformation matrix T.

2. For each slice i, i = 1 to n

  I. For each voxel *v* in slice i

   (a) Transform *v* to the atlas domain using the transformation matrix T.

   (b) Initialize a 3D cube, *C*, of size *N*_1*i*_ × *N*_2*i*_ × *N*_3*i*_ centered around the mapped voxel (*v*_mapped_).

   (c) Search *C* for voxels with corresponding grey level in all training sets with equalized intensities that fall within a predefined tolerance ±*τ* in *w*.

   (d) If no voxels are found using Step (c), increase size of *C* and repeat step (c) until correspondences are found or the maximum size allowed for *C* is reached.

   (e) Calculate the shape probability for each structure at location *r* based on the found voxels and their labels.

   End for

  End for

3. Return the constructed 4D shape probabilities.

#### First-order intensity model *P*(g|m)

The first-order visual appearance of each brain label is modeled by separating a mixed distribution of voxel intensities of the brain scans into individual components associated with the dominant modes of the mixture. The latter is precisely approximated with a Linear Combinations of Discrete Gaussians (LCDG) [63] with positive and negative components, which is based on a modified version of the classical Expectation-Maximization (EM) algorithm.

**Fig 4:**
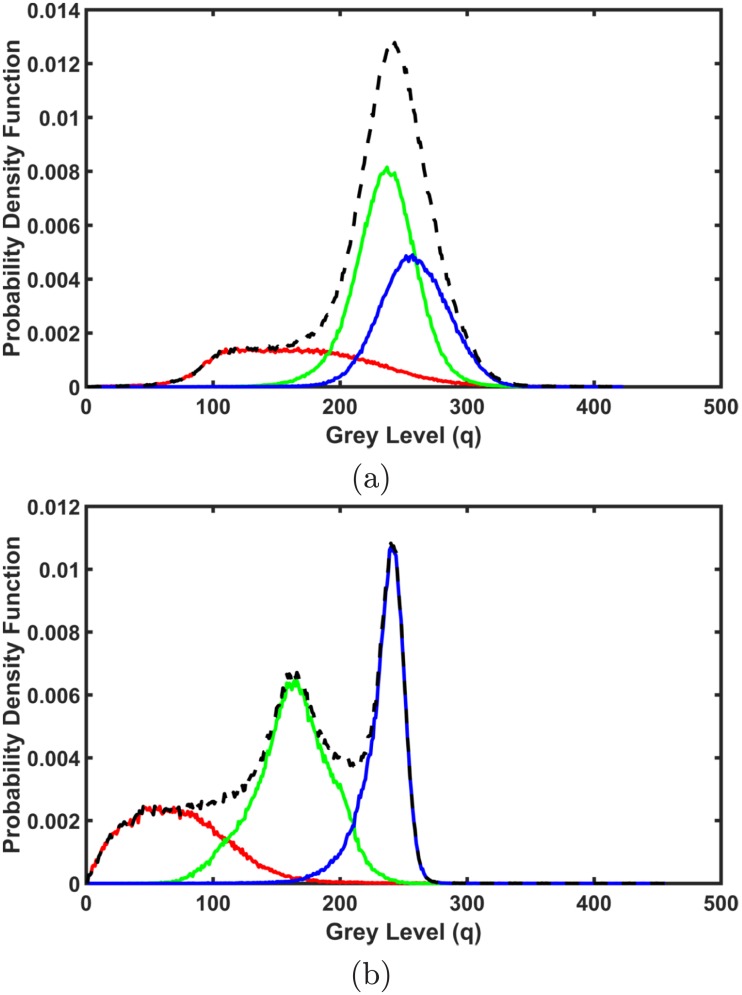
Normalized empirical density using the LCDG model for an infant subject (a), and an adult one (b). Note that dashed = empirical, red = CSF component, green = GM component, blue = WM component.

Let **Ψ**_*θ*_ = (*ψ*(*q*|*θ*) : *q* ∈ **Q**) denote a discrete Gaussian (DG) with parameters *θ* = (*μ*, *σ*), integrating a continuous 1D Gaussian density with mean *μ* and variance *σ*^2^ over successive gray level intervals. The LCDG with four dominant positive DGs and *C*_p_ ≥ 4 positive and *C*_n_ ≥ 0 negative subordinate DGs is [63]:

$$\begin{array}{c} P_{w,\Theta }\left(q\right)=\sum_{k=1}^{C_{p}}w_{p:k}\psi \left(q|\theta_{p:k}\right)-\sum_{\kappa =1}^{C_{n}}w_{n:\kappa }\psi \left(q|\theta_{n:\kappa }\right) \end{array}$$ (2)

where all the weights **w** = [*w*_p:*k*_, *w*_n:*κ*_] are non-negative and meet an obvious constraint $\sum_{k=1}^{C_{p}}w_{p:k}-\sum_{\kappa =1}^{C_{n}}w_{n:\kappa }=1$. All LCDG parameters, including *C*_p_ and *C*_n_, are estimated from the mixed empirical distribution to be modeled using the modified EM algorithm [63]. Fig 4 shows an example of the empirical density for different brain tissues using the LCDG model for an infant subject, Fig 4(a), and an adult one Fig 4(b).

#### MGRF model with second- and higher–order cliques *P*_V_(m)

**Fig 5:**
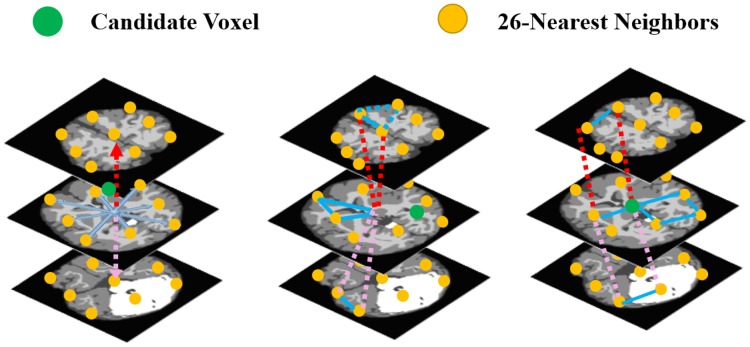
Samples of the second- (*a*), third- (*b*), and fourth-order (*c*) cliques for the 26-neighborhood (graph cliques are shown in different colors for visualization purpose).

In addition to the first-order visual appearance model, the spatial interactions between the brain voxels are also taken into account. Using spatial models that are only second-order-clique based, (e.g., [64]), will not enable accounting for the spatial inhomogeneity of brain MR images, especially for infants. Therefore, in this paper we propose a higher-order Markov-Gibbs Random Field (MGRF) spatial interaction model that adds the families of the triple and quad cliques to the pairwise cliques (Fig 5(b) and 5(c)), along with analytical estimation of the potentials. The proposed approach accounts for the spatial inhomogeneity of the brain scans, especially for those of infants, thus, reducing noise effects and increasing segmentation accuracy. Details of the proposed higher-order MGRF model are described below.

Let **C**_*a*_ denote a family of *s*-order cliques of an interaction graph with nodes in the 3D lattice sites (*x*, *y*, *z*) and edges connecting the interacting, or interdependent, sites (see Fig 5). To account for the scan inhomogeneities, especially with infant MRI, the label interactions are modeled by a spatially homogeneous MGRF with up to fourth-order interactions over the nearest 26-neighborhoods of voxels:

$$\begin{array}{c} P_{V}\left(m\right)=\frac{1}{Z_{V}}\exp(\sum_{a=1}^{A}\sum_{c\in C_{a}}V_{a}\left(m\left(x,y,z\right):\left(x,y,z\right)\in c\right)) \end{array}$$ (3)

where *A* clique families describe the geometry of the graph interactions, **V** = [*V*_*a*_ : {0, …, *L*} → (−∞, ∞) : *a* = 1, …, *A*] is a collection of Gibbs potential functions *V*_*a*_ for the families **C**_*a*_, and the partition function *Z*_**V**_ normalizes the probabilities over the parent population $M={\left\{0,\ldots ,L\right\}}^{XYZ}$ of all maps. An initial region map **m**, obtained by the voxel-wise classification, allows for analytically approximating the maximum likelihood estimates of the potentials and computing the voxel-wise probabilities of the region labels. For symmetry sake, only equality or inequality of the labels in clique **c** is taken into account. The second- third- and forth–order potentials are given by Eqs (4), (5) and (6), respectively:

Va(mp1,mp2)={V2:a:eqifmp1=mp2-V2:a:eqotherwise (4)

where $V_{2:a:\text{eq}}=-V_{2:a:\text{ne}}=4\;(F_{a:\text{eq}}\left(m{}^{\circ}\right)-\frac{1}{2})$, and **F**(**m**°) = [*ρ*_*a*_*F*_*a*_(*μ*_1_, …, *μ*_*s*_|**m**°) : (*μ*_1_, …, *μ*_*s*_) ∈ {0, …, *L*}^*s*^; *a* = 1, …, *A*] is the collection of scaled relative frequencies of co-occurrences of configurations (*μ*_1_, …, *μ*_*s*_) of the labels in the cliques of each family **C**_*a*_ over a given training map **m**°.

Va(mp1,mp2,mp3)={V3:a:eq3ifmp1=mp2=mp3-V3:a:eq3otherwise (5)

where $V_{3:a:{\text{eq}}_{3}}=-V_{3:a:{\text{eq}}_{2}}=\frac{16}{3}\;(F_{a:{\text{eq}}_{3}}\left(m{}^{\circ}\right)-\frac{1}{4})$

$$\begin{array}{c} V_{a}\;(m_{p_{1}},m_{p_{2}},m_{p_{3}},m_{p_{4}})=\{\begin{array}{cc} V_{4:a:{\text{eq}}_{4}} & \;\;\;\text{if}\;4\;\text{equal}\;\text{labels}\\ V_{4:a:{\text{eq}}_{3}} & \;\;\;\text{if}\;3\;\text{equal}\;\text{labels}\\ -(V_{4:a:{\text{eq}}_{3}}+V_{4:a:{\text{eq}}_{4}}) & \;\;\;\text{otherwise} \end{array} \end{array}$$ (6)

where

$$\begin{array}{ll} V_{4:a:{\text{eq}}_{4}} & =\lambda^{*}\left(F_{a:{\text{eq}}_{4}}(m{}^{\circ})-\frac{1}{8}\right)\\ V_{4:a:{\text{eq}}_{3}} & =\lambda^{*}\left(F_{a:{\text{eq}}_{3}}(m{}^{\circ})-\frac{1}{2}\right)\\ V_{4:a:{\text{eq}}_{2}} & =\lambda^{*}\left(F_{a:{\text{eq}}_{2}}(m{}^{\circ})-\frac{3}{8}\right)=-\left(V_{4:a:{\text{eq}}_{4}}+V_{4:a:{\text{eq}}_{3}}\right) \end{array}$$

and

$$\lambda^{*}=\frac{\sum_{a=1}^{A}\;\left({\left(F_{a:{\text{eq}}_{4}}\left(m{}^{\circ}\right)-\frac{1}{8}\right)}^{2}+{\left(F_{a:{\text{eq}}_{3}}\left(m{}^{\circ}\right)-\frac{1}{2}\right)}^{2}+{\left(F_{a:{\text{eq}}_{2}}\left(m{}^{\circ}\right)-\frac{3}{8}\right)}^{2}\right)}{\sum_{a=1}^{A}\;\left(\frac{7}{64}\;{\left(F_{a:{\text{eq}}_{4}}\left(m{}^{\circ}\right)-\frac{1}{8}\right)}^{2}+\frac{1}{4}\;{\left(F_{a:{\text{eq}}_{3}}\left(m{}^{\circ}\right)-\frac{1}{2}\right)}^{2}+\frac{15}{64}\;{\left(F_{a:{\text{eq}}_{2}}\left(m{}^{\circ}\right)-\frac{3}{8}\right)}^{2}\right)}$$

where *m*_**p**_*i*__ is the region map label at the voxel **p**_*i*_ = (*x*_*i*_, *y*_*i*_, *z*_*i*_). The proposed analytical approximation of the Gibbs potentials from a given map **m** extends earlier second-order MGRFs (e.g., [64]) to the higher-order models. The complete proof for the higher-order MGRF model is provided in the supplement.

Finally, the region map **m** is improved using Iterative Conditional Mode (ICM) algorithm [65] that maximizes the probabilities of the 3D joint model. The complete steps of our segmentation framework are summarized in Algorithm 2. Also Fig 2 illustrates the whole proposed framework.

## Experimental results

The proposed segmentation framework was tested on different databases at different ages to show its generality and robustness. 42 subjects from the Kennedy Krieger Institute (KKI)(8–12.8 years), 20 subjects from the university of California (UCLA) (8.4–17.9 years), and 20 subjects from the NYU Langone Medical Center (6.5–39.1 years) [66] were used to validate the segmentation approach. Moreover, 20 infants from the NDAR/IBIS database (aged 6 months) [67] were segmented using the proposed framework. For all subjects used throughout this study, there was no available information that would reveal the identity of the individual participants during or after data collection.

The IBIS database comprises T1-weighted images, and were acquired on a 3 tesla scanner with TR = 2400 millisecond (ms), TE = 3.16 ms TI = 1200 ms, and flip angle = 8. 160 sagittal slices were acquired at 1 millimeter (mm) thickness, with each slice being 224 × 256 pixels with 1 mm resolution.

The UCLA database includes participants between 8.4 and 17.9 years of age. T1-weighted images were acquired using MPRAGE with TR = 2300 ms, TE = 2.84 ms, and flip angle = 9. Sagittal slices were acquired at 1.2 mm thickness. The pixels of each 256 × 256 slice were 1 mm each side.

**Algorithm 2** Steps of the Proposed segmentation framework.

○ **MRI Preprocessing and Shape Database Construction**

  (a) Use the automated approach in [58] to remove the skull from the MR images.

  (b) Construct the shape database for each age group through a co-alignment of the biased-corrected training volumes (both grey scale and their ground truth).

○ **Brain Segmentation**

  (a) **For each Atlas**:

   i. Estimate the adaptive shape prior probability (*P*_*sp*_(**m**)) using Algorithm 1

   ii. Approximate *P*(**g**) using an LCDG with four dominant modes.

   iii. Form region map **m** using marginal estimated density and prior shape.

   iv. Find the Gibbs potentials for the MGRF model from the initial map **m**.

   v. Improve **m** using the iterative conditional mode (ICM) algorithm [65].

  (b) Apply majority voting to fuse the segmentation results of the three atlases.

Finally, the NYU data includes participants between 6.5 and 39.1 years of age. T1-weighted images were acquired on a 3 tesla Allegra with TR = 2530 ms, TE = 3.25 ms, and flip angle = 7. Sagittal slices were acquired at 1.33 mm thickness. The pixels of each 256 × 256 slice were 1.3 mm, and 1 mm. A summary of all the databases used in this work is provided in Table 2.

**Table 2:** Summary of databases used to validate the proposed method.

Database	Subjects	Age	Scan Parameters
IBIS	20	6 months	*B* = 3 T, TR = 2400 ms, TE = 3.16 ms flip angle = 8°, 1 mm thickness.
KKI	42	8–13 years	*B* = 3 T, TR = 8 ms, TE = 3.7 ms, flip angle = 8°, 1.33 mm thickness.
UCLA	20	8.4–17.9 years	TR = 2300 ms, TE = 2.84 ms, flip angle = 9°, 1.2 mm thickness.
NYU	20	6.5–39.1 years	*B* = 3 T, TR = 2530 ms, TE = 3.25 ms, flip angle = 7°, 1.33 mm thickness.

The proposed segmentation approach was evaluated on all subjects above using their manually segmented ground truth created by an MR expert. Special care was given to the infant subjects, since these were the hardest to delineate due to the unmyelinated nature of WM at this early age. Results in the upcoming pages show that the joint model combining the intensity, spatial, and shape information is in general a better performer than having only one or two of three models. The intensity information alone would often fail to differentiate between different tissue types of the infant MRI scans. This is enhanced after using higher-order MGRF, where edges of different tissue types are better retained. Comparing the visual segmentation results of using the intensity model alone for the IBIS database, with those for other databases, it can be inferred that major parts of the CWM bundles were missing for infant scans, which was not the case for the other databases. This result is expected, since the contrast is extremely poor between the different tissue types in infant MRI scans, whereas it gets better with older ages represented by the other databases. Results also show the merit of using the higher-order MGRF model with infant scans, which was compared against the second-order model and achieved better results. The performance of the two MGRF models with other age groups didn’t show a notable difference, which is expected since those scans are more homogeneous and less noisy than infant ones.

**Fig 6:**
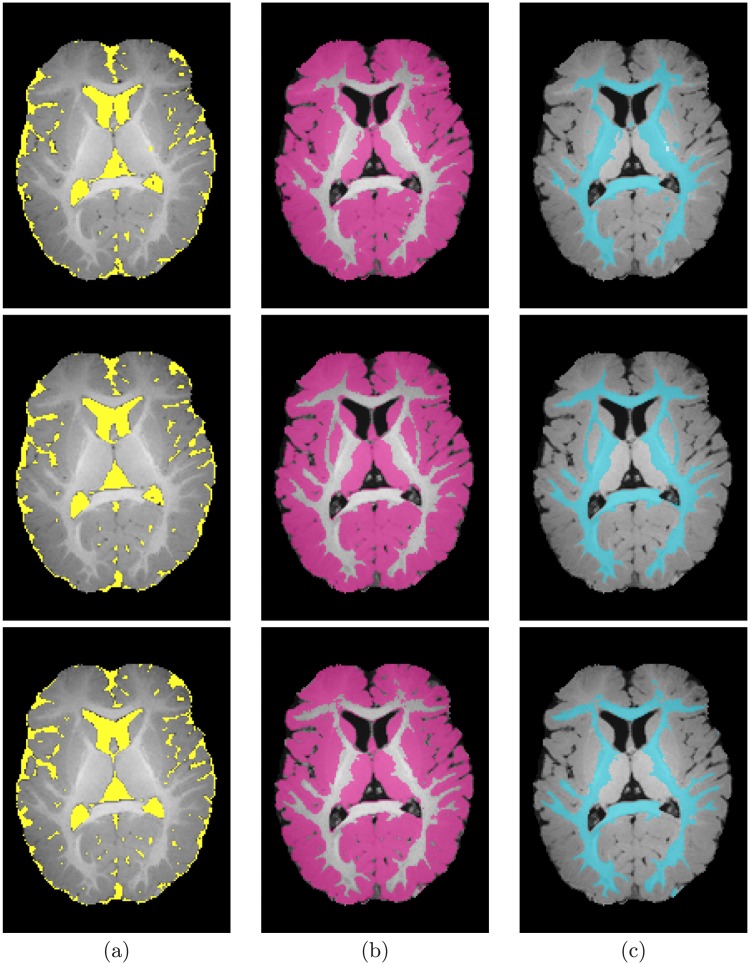
Segmentation results; for CSF (a), GM (b), and WM (c); projected onto axial plane for a six-month-old subject from the IBIS database for infants: Segmentation using our proposed method (*first row*); using the iBEAT method (*second row*); and Ground truth (*third row*).

**Fig 7:**
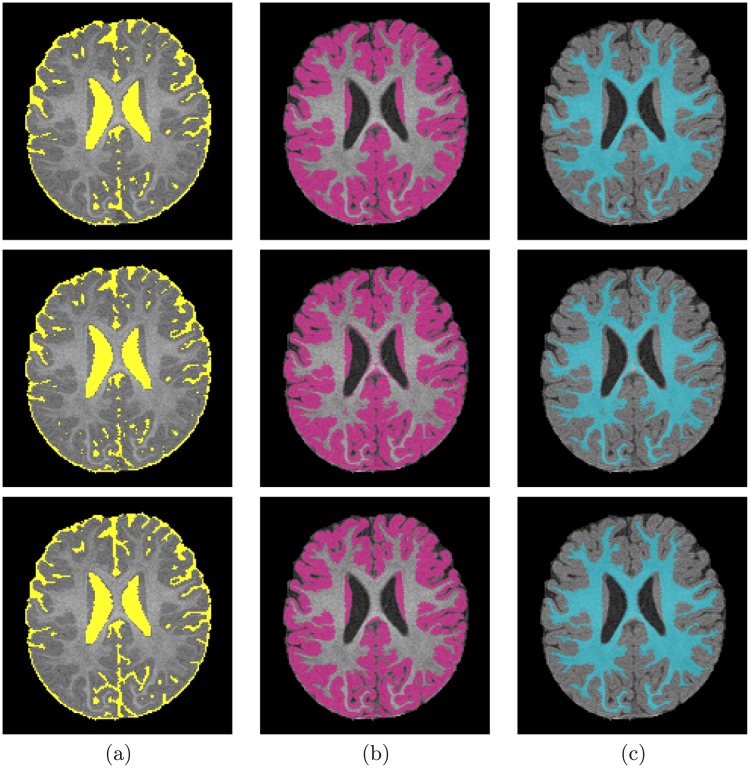
Segmentation results; for CSF (a), GM (b), and WM (c); projected onto axial plane for a additional six-month-old subject from the IBIS database for infants: Segmentation using our proposed method (*first row*); using the iBEAT method (*second row*); and Ground truth (*third row*).

**Fig 8:**
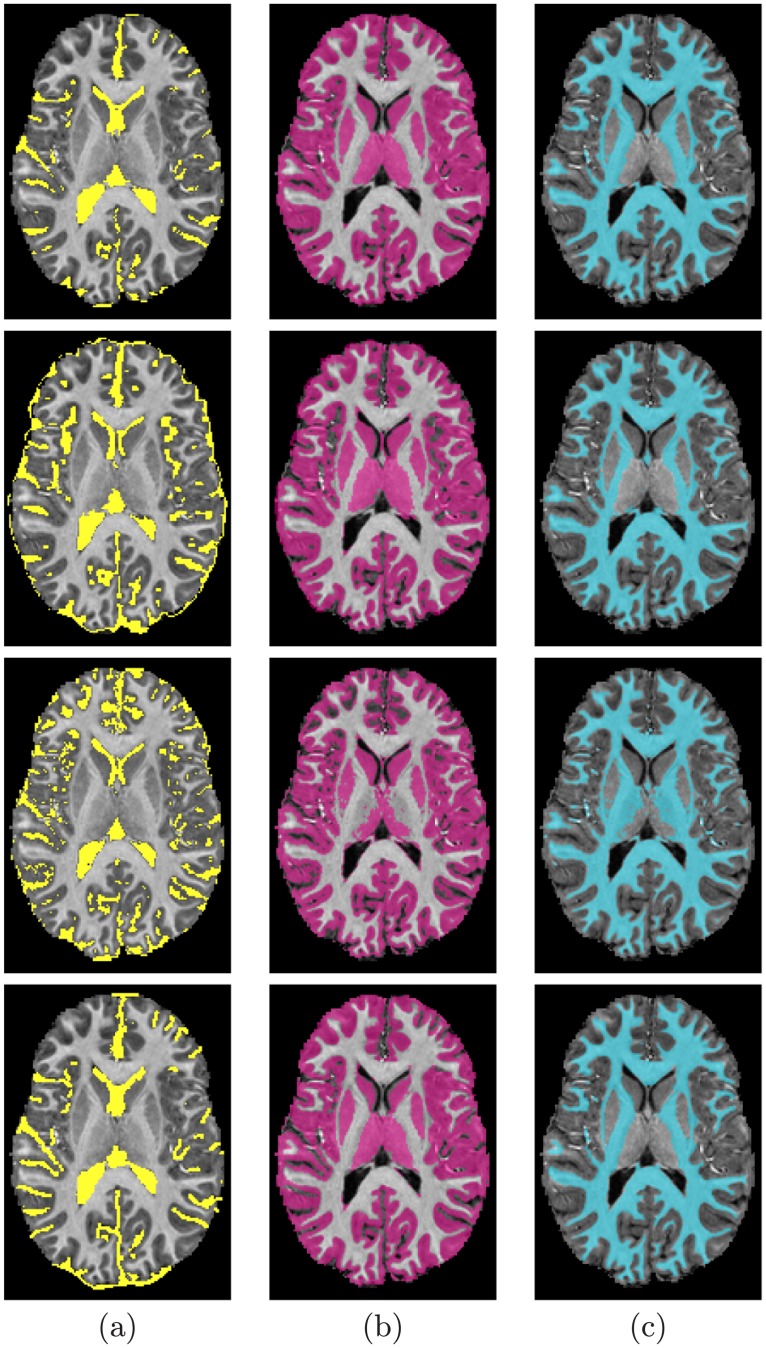
Segmentation results; for CSF (a), GM (b), and WM (c); projected onto axial plane for a nine-year-old subject from the KKI database: Segmentation using our proposed method (*first row*); using the FSL method (*second row*); using the FreeSurfer method (*third row*); and Ground truth (*fourth row*).

**Fig 9:**
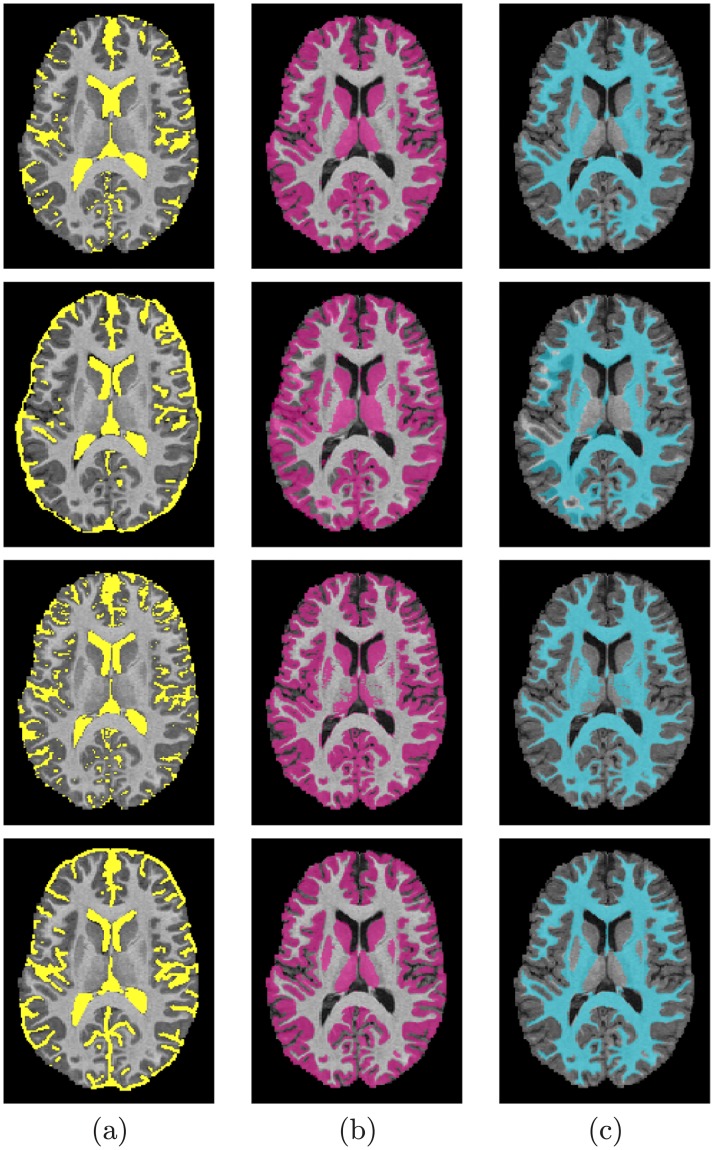
Segmentation results; for CSF (a), GM (b), and WM (c); projected onto axial plane for a 16-year-old subject from the UCLA database: Segmentation using our proposed method (*first row*); using the FSL method (*second row*); using the FreeSurfer method (*third row*); and Ground truth (*fourth row*).

**Fig 10:**
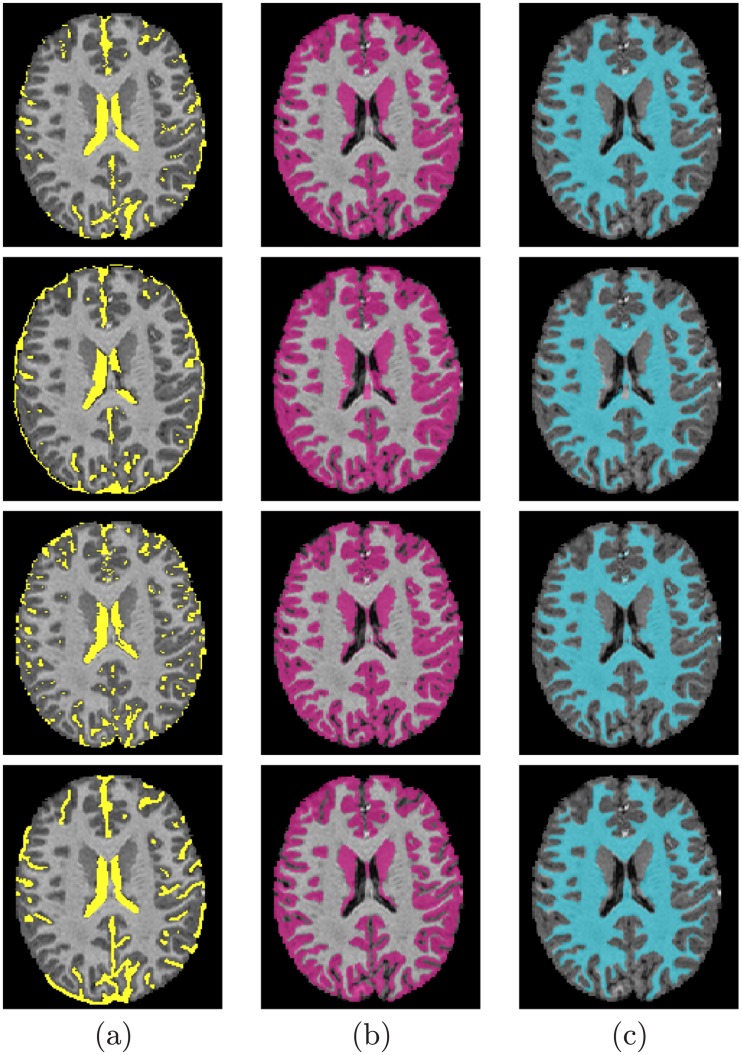
Segmentation results; for CSF (a), GM (b), and WM (c); projected onto axial plane for a sample from the NYU database: Segmentation using our proposed method (*first row*); using the FSL method (*second row*); using the FreeSurfer method (*third row*); and Ground truth (*fourth row*).

The segmentation performance was evaluated using 3 metrics: (*i*) the Dice similarity coefficient (DSC) [71], (*ii*) the 95-percentile modified Hausdorff distance (MHD) [72], and (*iii*) the absolute brain volume difference (ABVD), by comparing to the ground truth segmentation. Tables 3, 4, and 5 summarize the accuracy results obtained using the three metrics for the WM, GM, and CSF of different databases. As these tables show, the DSC metric was used to evaluate the proposed approach using both the second- and the higher-order MGRF. The reported accuracies show the advantages of using the higher-order MGRF, especially with the IBIS database, Table 3. Accuracies of 89.5% and 90.9% were achieved using the second-order model for WM and CGM segmentation respectively, whereas they enhanced to 94.7% and 95.2% with the higher-order model. The accuracies were also increased using the higher-order model with the other databases (Tables 4 and 5), yet not with the same rate it did with the IBIS database. This is acknowledged to the fact that the infant MR volumes suffer from more noise and image inhomogeneities which could be accounted for using the higher-order MGRF model. Segmentation results samples from each database are shown in Figs 6, 7, 8, 9 and 10, where the three extracted labels (WM, GM, CSF) using the proposed method and iBEAT [68], FSL [69], and FreeSurfer [70] are displayed along with the ground truth segmentation.

**Table 3:** Accuracy of our segmentation approach using Dice Similarity Coefficient (DSC)(%), the modified Hausdorff Distance (MHD)(mm), and Absolute Brain Volume Difference (ABVD) (%) for the WM, GM, and CSF of the IBIS database. Metrics are represented as Mean±Standard Deviation. Results for the proposed approach are shown using both the second- and higher-order MGRF model. Age of this group is 6 months.

		Segmentation Method				
Struct.	Metric	Proposed Method		iBEAT	FSL	FS
		2^nd^-order MGRF	Higher-order MGRF			
WM	DSC	89.5_±2.43_	94.7_±1.53_	73.3_±1.27_	80.4_±1.57_	85.6_±2.3_
	MHD	10.5_±0.7_	7.3_±1.23_	18.27_±1.53_	13.6_±1.28_	11.2_±1.0_
	ABVD	6.5_±2.54_	3.17_±1.73_	37.94_±0.61_	15.8_±0.6_	10.2_±3.7_
GM	DSC	90.9_±1.56_	95.2_±0.13_	81.6_±3.5_	89.5_±0.65_	86.5_±1.3_
	MHD	6.7_±1.5_	3.5_±0.24_	23.3_±0.52_	15.7_±0.86_	10.6_±3.7_
	ABVD	5.23_±3.1_	1.62_±1.24_	34.46_±0.18_	14.5_±1.17_	12.1_±2.5_
CSF	DSC	93.2_±1.2_	94.58_±0.44_	79.65_±1.38_	82.4_±1.5_	88.5_±1.4_
	MHD	5.6_±2_	4.35_±1.1_	27.23_±1.43_	9.7_±0.3_	7.8_±1_
	ABVD	2.5_±0.73_	1.9_±0.11_	21.07_±0.98_	7.5_±2_	5.3_±1.2_

**Table 4:** Accuracy of our segmentation approach using Dice Similarity Coefficient (DSC)(%), the modified Hausdorff Distance (MHD)(mm), and Absolute Brain Volume Difference (ABVD) (%) for the WM, GM, and CSF of the KKI database. Metrics are represented as Mean±Standard Deviation. Results for the proposed approach are shown using both the second- and higher-order MGRF model. Age range of this group is 8–13 years.

		Segmentation Method			
Struct.	Metric	Proposed Method		FSL	FS
		2^nd^-order MGRF	Higher-order MGRF		
WM	DSC	94.1_±0.8_	95.8_±1.5_	88.4_±2.5_	91.5_±2.3_
	MHD	5.8_±0.9_	5.2_±1.5_	9_±1.2_	7.5_±1.5_
	ABVD	3.2_±0.8_	2.5_±1.6_	9.8_±1.6_	9.1_±1.8_
GM	DSC	94.9_±0.77_	96.7_±1.2_	92.3_±1.85_	92.2_±1.2_
	MHD	4_±1.5_	3.1_±1.7_	11.8_±1.2_	8.2_±1.25_
	ABVD	1.8_±0.4_	1.12_±1.1_	8.3_±2.7_	5.2_±1.4_
CSF	DSC	95.5_±1.25_	96.5_±1_	92.1_±2.7_	93.2_±3.7_
	MHD	5_±1.3_	3.7_±2_	7.25_±2.1_	7_±1.5_
	ABVD	3_±1.25_	2.5_±1_	9_±2.0_	8.25_±2.1_

**Table 5:** Accuracy of our segmentation approach using Dice Similarity Coefficient (DSC)(%), the modified Hausdorff Distance (MHD)(mm), and Absolute Brain Volume Difference (ABVD) (%) for the WM, GM, and CSF of the NYU and UCLA databases. Metrics are represented as Mean±Standard Deviation. Results for the proposed approach are shown using both the second- and higher-order MGRF model. Age range of this group is 6.5–39.1 years.

		Segmentation Method			
Struct.	Metric	Proposed Method		FSL	FS
		2^nd^-order MGRF	Higher-order MGRF		
WM	DSC	95.7_±1.1_	96.1_±1.5_	92.4_±1.5_	93.5_±0.2_
	MHD	2.7_±0.5_	2.3_±1.2_	11.8_±1.8_	7.5_±2.0_
	ABVD	1.9_±0.72_	1.5_±1.7_	10.7_±1.6_	7.8_±2_
GM	DSC	97.1_±0.6_	97.8_±0.13_	93.2_±1.3_	94.4_±1.8_
	MHD	1.3_±0.44_	0.9_±0.2_	9.1_±1.6_	5_±0.8_
	ABVD	1.88_±0.76_	1.2_±1.4_	10.6_±1.73_	7_±2.3_
CSF	DSC	96.25_±1_	97.5_±0.7_	91.5_±2_	93_±1.5_
	MHD	2_±0.65_	1.5_±0.8_	8.5_±0.6_	7_±1.2_
	ABVD	1.5_±0.2_	1_±0.3_	9.5_±3_	8.2_±0.9_

The performance of the proposed segmentation approach is highlighted by comparing it against the software package (iBEAT) [68], that performs bias correction followed by brain segmentation. Moreover, segmentation was done using the FSL package [69], and also using FreeSurfer software [70]. Segmentation accuracies for the iBEAT, FSL, and FreeSurfer results are also summarized in Tables 3, 4, and 5, where accuracies are reported for CWM, GM, and CSF. These results emphasize the efficiency of the proposed approach that is required for possible subsequent processes such as shape analysis. Table 6 breaks down the timing expended by each approach to perform segmentation for a single subject. The proposed approach turned out to be the fastest, which adds up to its advantages.

**Table 6:** Summary of the time required by the proposed approach and other approaches for segmenting a brain subject.

Approach	Required execution time
iBEAT [68]	40 minutes
FSL [69]	22 minutes
FreeSurfer [70]	18 hours
Proposed approach	92 seconds for skull stripping, 370 seconds for alignment, 65 seconds for segmentation, Total is about 9 minutes.

It is worth mentioning that in some cases where the anatomical changes in the brain structures are irregular/rapid, there will be a lack of support from the 3D relative neighbors which will affect the 3D spatial dependencies between each voxels and its neighbors. This smoothness assumption by the MGRF model may results in an underestimation of thin white matter strands. Fig 6 shows an example of such effect for an axial cross section from one subject from the IBIS database, while Fig 7 shows another axial cross section of enhanced results.

## Conclusions and future work

This paper proposed a 3D automated approach for brain segmentation from MR images in subjects spanning different ages. The segmentation method integrates intensity, shape, and spatial information in a hybrid model. The MGRF spatial model, the higher-order one in particular, accounts for scan inhomogeneities and noise that are drastically affecting infant scans. The novelty of the proposed algorithm lies at using the adaptive shape model along with the higher-order MGRF model. The work has been tested and validated on 102 MR scans and compared against state-of-the-art approaches. The metrics used to evaluate the segmentation showed that the proposed approach is a better performer in terms of accuracy and time.

## References

[1] Balafar MA, Ramli AR, Saripan MI, Mashohor S. Review of brain MRI image segmentation methods. Artif Intell Rev. 2010;33(3):261–274. doi: 10.1007/s10462-010-9155-0

[2] Weisenfeld NI, Warfield SK. Automatic segmentation of newborn brain MRI. Neuroimage. 2009;47(2):564–572. doi: 10.1016/j.neuroimage.2009.04.068 19409502 10.1016/j.neuroimage.2009.04.068 PMC2945911

[3] El-Baz A, Gimel’farb G, Suri JS. Stochastic modeling for medical image analysis. CRC Press; 2015.

[4] Mewes AUJ, Hüppi PS, Als H, Rybicki FJ, Inder TE, McAnulty GB, et al Regional brain development in serial magnetic resonance imaging of low-risk preterm infants. Pediatrics. 2006;118(1):23–33. doi: 10.1542/peds.2005-2675 16818545 10.1542/peds.2005-2675

[5] Xue H, Srinivasan L, Jiang S, Rutherford M, Edwards AD, Rueckert D, et al Automatic segmentation and reconstruction of the cortex from neonatal MRI. Neuroimage. 2007;38(3):461–477. doi: 10.1016/j.neuroimage.2007.07.030 17888685 10.1016/j.neuroimage.2007.07.030

[6] Barkovich AJ. Magnetic resonance techniques in the assessment of myelin and myelination. J Inherited Metab Dis. 2005;28(3):311–343. doi: 10.1007/s10545-005-5952-z 15868466 10.1007/s10545-005-5952-z

[7] Ng H, Ong S, Foong K, Goh P, Nowinski W. Medical image segmentation using K-means clustering and improved watershed algorithm. In: Image Analysis and Interpretation, 2006 IEEE Southwest Symposium on. IEEE; 2006. p. 61–65.

[8] Mayer A, Greenspan H. An adaptive mean-shift framework for MRI brain segmentation. IEEE Trans Med Imag. 2009;28(8):1238–1250. doi: 10.1109/TMI.2009.2013850 10.1109/TMI.2009.2013850 19211339

[9] Fang R, Chen YhJ, Zabih R, Chen T. Tree-metrics graph cuts for brain MRI segmentation with tree cutting. In: Image Processing Workshop (WNYIPW), 2010 Western New York. IEEE; 2010. p. 10–13.

[10] Ortiz A, Górriz J, Ramirez J, Salas-Gonzalez D. MR brain image segmentation by growing hierarchical SOM and probability clustering. Electron Lett. 2011;47(10):585–586. doi: 10.1049/el.2011.0322

[11] Li L, Xie M, Gao J, Yue X. MRI Brain Segmentation Based on a Three-Dimensional Markov Random Field Model In: Unifying Electrical Engineering and Electronics Engineering. Springer; 2014 p. 1233–1239.

[12] Janney JB, Aarthi A, Reddy SRK. An Automatic MRI Brain Segmentation by Using Adaptive Mean-Shift Clustering Framework. In: Proceedings of International Conference on Internet Computing and Information Communications. Springer; 2014. p. 111–119.

[13] Weber J, Doenitz C, Brawanski A, Palm C. Data-Parallel MRI Brain Segmentation in Clinical Use In: Bildverarbeitung für die Medizin 2015. Springer; 2015 p. 389–394.

[14] Mahmood Q, Chodorowski A, Persson M. Automated MRI brain tissue segmentation based on mean shift and fuzzy c-means using a priori tissue probability maps. IRBM. 2015;36(3):185–196. doi: 10.1016/j.irbm.2015.01.007

[15] Anbeek P, Vincken KL, Groenendaal F, Koeman A, Van Osch MJ, Van der Grond J. Probabilistic brain tissue segmentation in neonatal magnetic resonance imaging. Pediatr Res. 2008;63(2):158–163. doi: 10.1203/PDR.0b013e31815ed071 18091357 10.1203/PDR.0b013e31815ed071

[16] Wang L, et al LINKS: Learning-based Multi-source IntegratioN frameworK for Segmentation of Infant Brain Image. Neuroimage. 2015;108:160–172. doi: 10.1016/j.neuroimage.2014.12.042 25541188 10.1016/j.neuroimage.2014.12.042 PMC4323750

[17] Zhang W, Li R, Deng H, Wang L, Lin W, Ji S, et al Deep convolutional neural networks for multi-modality isointense infant brain image segmentation. Neuroimage. 2015;108:214–224. doi: 10.1016/j.neuroimage.2014.12.061 25562829 10.1016/j.neuroimage.2014.12.061 PMC4323729

[18] Moeskops P, Benders MJ, Chiţa SM, Kersbergen KJ, Groenendaal F, de Vries LS, et al Automatic segmentation of MR brain images of preterm infants using supervised classification. Neuroimage. 2015;118:628–641. doi: 10.1016/j.neuroimage.2015.06.007 26057591 10.1016/j.neuroimage.2015.06.007

[19] Ashburner J, Friston KJ. Unified segmentation. Neuroimage. 2005;26(3):839–851. doi: 10.1016/j.neuroimage.2005.02.018 15955494 10.1016/j.neuroimage.2005.02.018

[20] Pohl KM, Fisher J, Grimson WEL, Kikinis R, Wells WM. A Bayesian model for joint segmentation and registration. Neuroimage. 2006;31(1):228–239. doi: 10.1016/j.neuroimage.2005.11.044 16466677 10.1016/j.neuroimage.2005.11.044

[21] Han X, Fischl B. Atlas renormalization for improved brain MR image segmentation across scanner platforms. IEEE Trans Med Imag. 2007;26(4):479–486. doi: 10.1109/TMI.2007.893282 10.1109/TMI.2007.893282 17427735

[22] Artaechevarria X, Munoz-Barrutia A, Ortiz-de Solorzano C. Combination strategies in multi-atlas image segmentation: Application to brain MR data. IEEE Trans Med Imag. 2009;28(8):1266–1277. doi: 10.1109/TMI.2009.2014372 10.1109/TMI.2009.2014372 19228554

[23] Sabuncu MR, Yeo BT, Van Leemput K, Fischl B, Golland P. A generative model for image segmentation based on label fusion. IEEE Trans Med Imag. 2010;29(10):1714–1729. doi: 10.1109/TMI.2010.2050897 10.1109/TMI.2010.2050897 PMC3268159 20562040

[24] Morin JP, Desrosiers C, Duong L. Atlas-based segmentation of brain magnetic resonance imaging using random walks. In: Computer Vision and Pattern Recognition Workshops (CVPRW), 2012 IEEE Computer Society Conference on. IEEE; 2012. p. 44–49.

[25] Morel JM, Yu G. ASIFT: A new framework for fully affine invariant image comparison. SIAM J Imag Sci. 2009;2(2):438–469. doi: 10.1137/080732730

[26] Bay H, Tuytelaars T, Van Gool L. Surf: Speeded up robust features In: Computer Vision–ECCV 2006. Springer; 2006 p. 404–417.

[27] Lötjönen JM, Wolz R, Koikkalainen JR, Thurfjell L, Waldemar G, Soininen H, et al Fast and robust multi-atlas segmentation of brain magnetic resonance images. Neuroimage. 2010;49(3):2352–2365. doi: 10.1016/j.neuroimage.2009.10.026 19857578 10.1016/j.neuroimage.2009.10.026

[28] van der Lijn F, de Bruijne M, Klein S, den Heijer T, Hoogendam YY, van der Lugt A, et al Automated brain structure segmentation based on atlas registration and appearance models. IEEE Trans Med Imag. 2012;31(2):276–286. doi: 10.1109/TMI.2011.2168420 10.1109/TMI.2011.2168420 21937346

[29] Boykov Y, Veksler O, Zabih R. Fast approximate energy minimization via graph cuts. IEEE Trans Pattern Anal Mach Intell. 2001;23(11):1222–1239. doi: 10.1109/34.969114

[30] Ledig C, Wolz R, Aljabar P, Lotjonen J, Heckemann RA, Hammers A, et al. Multi-class brain segmentation using atlas propagation and EM-based refinement. In: Biomedical Imaging (ISBI), 2012 9th IEEE International Symposium on. IEEE; 2012. p. 896–899.

[31] Srhoj-Egekher V, Benders MJNL, Kersbergen KJ, Viergever MA, Isgum I. Automatic segmentation of neonatal brain MRI using atlas based segmentation and machine learning approach In: MICCAI Grand Challenge: Neonatal Brain Segmentation; 2012 p. 22–27.

[32] Wang L, Shi F, Yap PT, Lin W, Gilmore JH, Shen D. Longitudinally guided level sets for consistent tissue segmentation of neonates. Hum Brain Mapp. 2013;34(4):956–972. doi: 10.1002/hbm.21486 22140029 10.1002/hbm.21486 PMC4855279

[33] Shi F, Fan Y, Tang S, Gilmore JH, Lin W, Shen D. Neonatal brain image segmentation in longitudinal MRI studies. Neuroimage. 2010;49(1):391–400. doi: 10.1016/j.neuroimage.2009.07.066 19660558 10.1016/j.neuroimage.2009.07.066 PMC2764995

[34] Cherel M, Budin F, Prastawa M, Gerig G, Lee K, Buss C, et al; Automatic tissue segmentation of neonate brain MR Images with subject-specific atlases. Proc SPIE International Society for Optics; Photonics. 2015;9413:941311. 10.1117/12.2082209 PMC4469197 26089584

[35] Makropoulos A, Gousias IS, Ledig C, Aljabar P, Serag A, Hajnal JV, et al Automatic whole brain MRI segmentation of the developing neonatal brain. IEEE Trans Med Imag. 2014;33(9):1818–1831. doi: 10.1109/TMI.2014.2322280 10.1109/TMI.2014.2322280 24816548

[36] Altaye M, Holland SK, Wilke M, Gaser C. Infant brain probability templates for MRI segmentation and normalization. Neuroimage. 2008;43(4):721–730. doi: 10.1016/j.neuroimage.2008.07.060 18761410 10.1016/j.neuroimage.2008.07.060 PMC2610429

[37] Song Z, et al Clinical neonatal brain MRI segmentation using adaptive nonparametric data models and intensity-based Markov priors In: MICCAI. Springer; 2007 p. 883–890. 10.1007/978-3-540-75757-3_107 18051142

[38] Song Z, Tustison N, Avants B, Gee J. Adaptive graph cuts with tissue priors for brain MRI segmentation. In: Biomedical Imaging: Nano to Macro, 2006. 3rd IEEE International Symposium on. IEEE; 2006. p. 762–765.

[39] Song T, Jamshidi MM, Lee RR, Huang M. A modified probabilistic neural network for partial volume segmentation in brain MR image. IEEE Trans Neural Netw. 2007;18(5):1424–1432. doi: 10.1109/TNN.2007.891635 18220190 10.1109/tnn.2007.891635

[40] Patenaude B, Smith SM, Kennedy DN, Jenkinson M. A Bayesian model of shape and appearance for subcortical brain segmentation. Neuroimage. 2011;56(3):907–922. doi: 10.1016/j.neuroimage.2011.02.046 21352927 10.1016/j.neuroimage.2011.02.046 PMC3417233

[41] Serag A, Wilkinson AG, Telford EJ, Pataky R, Sparrow SA, Anblagan D, et al SEGMA: an automatic SEGMentation Approach for human brain MRI using sliding window and random forests. Front Neuroinform. 2017;11:2 doi: 10.3389/fninf.2017.00002 28163680 10.3389/fninf.2017.00002 PMC5247463

[42] Wang L, Shi F, Li G, Lin W, Gilmore JH, Shen D. Integration of Sparse Multi-modality Representation and Geometrical Constraint for Isointense Infant Brain Segmentation In: Medical Image Computing and Computer-Assisted Intervention–MICCAI 2013. Springer; 2013 p. 703–710. 10.1007/978-3-642-40811-3_88 PMC4066845 24505729

[43] Angelini ED, Song T, Mensh BD, Laine AF. Segmentation and quantitative evaluation of brain MRI data with a multi-phase three-dimensional implicit deformable model. Proc SPIE. 2004;5370:526.

[44] Colliot O, Camara O, Bloch I. Integration of fuzzy spatial relations in deformable models: Application to brain MRI segmentation. Patt Recogn. 2006;39(8):1401–1414.

[45] Miri S, Passat N, Armspach JP. Topology-preserving discrete deformable model: Application to multi-segmentation of brain MRI In: Image and Signal Processing. Springer; 2008 p. 67–75.

[46] Liu JX, Chen YS, Chen LF. Accurate and robust extraction of brain regions using a deformable model based on radial basis functions. J Neurosci Methods. 2009;183(2):255–266. doi: 10.1016/j.jneumeth.2009.05.011 19467263 10.1016/j.jneumeth.2009.05.011

[47] Albert Huang A, Abugharbieh R, Tam R. A Hybrid Geometric–Statistical Deformable Model for Automated 3-D Segmentation in Brain MRI. IEEE Trans Biomed Eng. 2009;56(7):1838–1848. doi: 10.1109/TBME.2009.2017509 19336280 10.1109/TBME.2009.2017509 PMC3068615

[48] Del Fresno M, Vénere M, Clausse A. A combined region growing and deformable model method for extraction of closed surfaces in 3D CT and MRI scans. Comput Med Imag Graph. 2009;33(5):369–376. doi: 10.1016/j.compmedimag.2009.03.002 10.1016/j.compmedimag.2009.03.002 19346100

[49] Wang L, Chen Y, Pan X, Hong X, Xia D. Level set segmentation of brain magnetic resonance images based on local Gaussian distribution fitting energy. J Neurosci Methods. 2010;188(2):316–325. doi: 10.1016/j.jneumeth.2010.03.004 20230858 10.1016/j.jneumeth.2010.03.004

[50] Bourouis S, Hamrouni K. 3D segmentation of MRI brain using level set and unsupervised classification. Int J Image Graph. 2010;10(01):135–154. doi: 10.1142/S0219467810003706

[51] Ciofolo C, Barillot C. Atlas-based segmentation of 3D cerebral structures with competitive level sets and fuzzy control. Med Image Anal. 2009;13(3):456–470. doi: 10.1016/j.media.2009.02.008 19362876 10.1016/j.media.2009.02.008

[52] Wang XH, Liu B, Song ZQ. 3-Dimensional Brain MRI Segmentation Based on Multi-Layer Background Subtraction and Seed Region Growing Algorithm In: Applied Mechanics and Materials. vol. 536 Trans Tech Publ; 2014 p. 218–221.

[53] Zhao M, Lin HY, Yang CH, Hsu CY, Pan JS, Lin MJ. Automatic threshold level set model applied on MRI image segmentation of brain tissue. Appl Math. 2015;9(4):1971–1980.

[54] de Brebisson A, Montana G. Deep neural networks for anatomical brain segmentation. In: Proceedings of the IEEE Conference on Computer Vision and Pattern Recognition Workshops; 2015. p. 20–28.

[55] Chen H, Dou Q, Yu L, Heng PA. Voxresnet: Deep voxelwise residual networks for volumetric brain segmentation. arXiv preprint arXiv:160805895. 2016;. 10.1016/j.neuroimage.2017.04.041 28445774

[56] Tustison NJ, Avants BB, Cook PA, Zheng Y, Egan A, Yushkevich PA, et al N4ITK: improved N3 bias correction. IEEE Trans Med Imag. 2010;29(6):1310–1320. doi: 10.1109/TMI.2010.2046908 10.1109/TMI.2010.2046908 PMC3071855 20378467

[57] Bouman C, Sauer K. A generalized Gaussian image model for edge-preserving MAP estimation. IEEE Trans Image Process. 1993;2(3):296–310. doi: 10.1109/83.236536 18296219 10.1109/83.236536

[58] Alansary A, Ismail M, Soliman A, Khalifa F, Nitzken M, Elnakib A, et al Infant Brain Extraction in T1-weighted MR Images using BET and Refinement using LCDG and MGRF Models. IEEE J Biomed Health Inform. 2016;20(3):925–935. doi: 10.1109/JBHI.2015.2415477 25823048 10.1109/JBHI.2015.2415477

[59] Viola PA, Wells WM III. Alignment by Maximization of Mutual Information. Int J Comput Vis. 1997;24(2):137–154.

[60] Ismail M, Soliman A, ElTanboly A, Switala A, Mahmoud M, Khalifa F, et al. Detection of White Matter Abnormalities In MR Brain Images for Diagnosis of Autism in Children. In: Biomedical Imaging: From Nano to Macro, 2011 IEEE International Symposium on. IEEE; 2016. p. 6–9.

[61] Ismail M, Mostapha M, Soliman A, Nitzken M, Khalifa F, Elnakib A, et al. Segmentation of infant brain MR images based on adaptive shape prior and higher-order MGRF. In: Image Processing (ICIP), 2015 IEEE International Conference on. IEEE; 2015. p. 4327–4331.

[62] Soliman A, Khalifa F, Elnakib A, El-Ghar MA, Dunlap N, Wang B, et al Accurate lungs segmentation on CT chest images by adaptive appearance-guided shape modeling. IEEE transactions on medical imaging. 2017;36(1):263–276. doi: 10.1109/TMI.2016.2606370 27705854 10.1109/TMI.2016.2606370

[63] El-Baz A, et al Precise segmentation of 3-D magnetic resonance angiography. IEEE Trans Biomed Eng. 2012;59(7):2019–2029. doi: 10.1109/TBME.2012.2196434 22547453 10.1109/TBME.2012.2196434

[64] Farag A, El-Baz A, Gimel’farb G. Precise segmentation of Multimodal Images. IEEE Trans Image Process. 2006;15(4):952–968. doi: 10.1109/TIP.2005.863949 16579381 10.1109/tip.2005.863949

[65] Besag J. On the statistical analysis of dirty pictures. J R Stat Soc B. 1986;48(3):259–302.

[66] Di Martino A, Yan CG, Li Q, Denio E, Castellanos FX, Alaerts K, et al The autism brain imaging data exchange: towards a large-scale evaluation of the intrinsic brain architecture in autism. Mol Psychiatry. 2014;19(6):659–667. doi: 10.1038/mp.2013.78 23774715 10.1038/mp.2013.78 PMC4162310

[67] Hall D, Huerta MF, McAuliffe MJ, Farber GK. Sharing heterogeneous data: the National Database for Autism Research. Neuroinformatics. 2012;10(4):331–339. doi: 10.1007/s12021-012-9151-4 22622767 10.1007/s12021-012-9151-4 PMC4219200

[68] Yakang D, Shi F, Wang L, Wu G, Shen D. iBEAT: a toolbox for infant brain magnetic resonance image processing. Neuroinformatics. 2013;11(2):211–225. doi: 10.1007/s12021-012-9164-z 23055044 10.1007/s12021-012-9164-z

[69] Jenkinson M, Beckmann CF, Behrens TEJ, Woolrich MW, Smith SM. Fsl. Neuroimage. 2012;62(2):782–790. doi: 10.1016/j.neuroimage.2011.09.015 21979382 10.1016/j.neuroimage.2011.09.015

[70] Fischl B. FreeSurfer. Neuroimage. 2012;62(2):774–781. doi: 10.1016/j.neuroimage.2012.01.021 22248573 10.1016/j.neuroimage.2012.01.021 PMC3685476

[71] Dice LR. Measures of the amount of ecologic association between species. Ecology. 1945;26:297–302. doi: 10.2307/1932409

[72] Gerig G, Jomier M, Chakos M. Valmet: A new validation tool for assessing and improving 3D object segmentation In: MICCAI; 2001 p. 516–523.

